# Antioxidant activity of the probiotic consortium in vitro

**DOI:** 10.5195/cajgh.2013.115

**Published:** 2014-01-24

**Authors:** Saule Saduakhasova, Almagul Kushugulova, Samat Kozhakhmetov, Gulnara Shakhabayeva, Indira Tynybayeva, Talgat Nurgozhin, Zhaxybay Zhumadilov

**Affiliations:** Center for Life Sciences, Nazarbayev University

**Keywords:** antioxidant activity, probiotic bacteria

## Abstract

**Introduction:**

Available evidence suggests that probiotics have different biological functions that depend on several mechanisms, such as antioxidant and DNA-protective activities. The probiotic consortium includes bacterial cultures such as *Streptococcus thermophilus, Lactococcus lactis, Lactobacillus plantarum*, and other bacterial cultures isolated from traditional Kazakh dairy products (ayran, kumys, shubat, and healthy clinical material). The aim of this study was to investigate the total antioxidant activity of the consortium of probiotic bacteria and to determine the activity of superoxide dismutase, glutathione reductase, and DNA-protective action.

**Material and methods:**

In vitro comet assay was used to determine the antigenotoxicity of the probiotic consortium. Total antioxidant activity was determined using a method of analysis with Trolox as the equivalent. The analysis method of superoxide dismutase activity assesses the inhibition rate of the nitroblue tetrazolium reduction to formazan by superoxide dismutase. Determination of glutathione reductase activity is based on the measurement of the NADPH oxidation speed.

**Results:**

A significantly high level of the total antioxidant activity of the probiotic consortium intact cells (15.3 mM/ml) was observed whereas the activity index of lysate was 11.1 mM/ml.

The superoxide dismutase activity of probiotic consortium lysate was evaluated, with values that peaked at 0.24 U/mg protein. The superoxide dismutase activity of the consortium was lower in comparison to L.fernentum E-3 and L.fernentum E-18 cultures with values of 0.85 U/mg and 0.76 U/mg protein, respectively. SOD activity of probiotic consortium whole cells was not observed, which is typical for lactic acid bacteria.

Glutathione reductase plays an important role in the optimal protection from oxidative stress. Glutathione reductase activity of the studied probiotic consortium was low; moreover, the activity of the lysate was two times higher than the activity of the cells reaching 0.01 units/ml. Investigations by Dr. Li have shown that the intracellular glutathione may give a significant protection of Lactococcus from the damaging action of H_2_O_2_, even at very low concentrations.

The data from our study suggests that the co-incubation of the epithelial cells with probiotic bacteria reduces the percentage of damaged cells (damage index–0.60).

**Conclusion:**

The studied probiotic consortium has antigenotoxic and antioxidant activities. Preparations and products of this probiotic consortium may serve as a protective component in the intestinal microbial ecosystem.

